# Zirconia versus Titanium Implants: 8-Year Follow-Up in a Patient Cohort Contrasted with Histological Evidence from a Preclinical Animal Model

**DOI:** 10.3390/ma15155322

**Published:** 2022-08-02

**Authors:** Warwick J. Duncan, Sunyoung Ma, Allauddin Siddiqi, Reham B. Osman

**Affiliations:** 1 Faculty of Dentistry, University of Otago, P.O. Box 56, Dunedin 9016, New Zealand; sunyoung.ma@otago.ac.nz; 2 Specialist Private Practice, Brisbane, QLD 4122, Australia; siddiqidr@gmail.com; 3 Faculty of Oral and Dental Medicine, Cairo University, Cairo 12613, Egypt; rehambosman@gmail.com

**Keywords:** animal trial, dental implants, histomorphometry, bone-implant contact

## Abstract

Zirconia ceramic (ZC) implants are becoming more common, but comparisons between preclinical histology and long-term clinical trials are rare. This investigation comprised (1) 8-year clinical follow-up of one-piece ZC or titanium (Ti) implants supporting full overdentures and (2) histomorphometric analysis of the same implants in an animal model, comparing implants with various surface treatments. Methods: (1) Clinical trial: 24 completely edentulous participants (2 groups of N = 12) received 7 implants (one-piece ball-abutment ZC or Ti; maxilla N = 4, mandible N = 3) restored with implant overdentures. Outcomes after 8-years included survival, peri-implant bone levels, soft-tissue responses, and prosthodontic issues. (2) Preclinical trial: 10 New Zealand sheep received 4 implants bilaterally in the femoral condyle: Southern Implants ZC or Ti one-piece implants, identical to the clinical trial, and controls: Southern ITC^®^ two-piece implants with the same surface or Nobel (NBC) anodised (TiUnite™) surface. %Bone-implant contact (%BIC) was measured after 12 weeks of unloaded healing. Results: 8 of 24 participants (33%) of an average age of 75 ± 8 years were recalled; 21% of original participants had died, and 46% could not be contacted. 80.4% of implants survived; excluding palatal sites, 87.5% of Ti and 79% of ZC implants survived. All failed implants were in the maxilla. Three ZC implants had fractured. Bone loss was similar for Ti vs. ZC; pocket depths (*p* = 0.04) and attachment levels (*p* = 0.02) were greater for Ti than ZC implants. (1.7 ± 1.6 mm vs. 1.6 ± 1.3 mm). All implants in sheep femurs survived. %BIC was not statistically different for one-piece blasted surface Ti (80 ± 19%) versus ZC (76 ± 20%) or ITC^®^ (75 ± 16 mm); NBC had significantly higher %BIC than ITC (84 ± 17%, *p* = 0.4). Conclusion: Short-term preclinical results for ZC and Ti one-piece implants showed excellent bone-implant contact in unloaded femoral sites. This differed from the long-term clinical results in older-aged, edentulous participants. While ZC and Ti implants showed equivalent performance, the risks of peri-implantitis and implant loss in older, completely edentulous patients remain a significant factor.

## 1. Introduction

Metal-free restoration of teeth and dental implants have become popular due to their improved aesthetics, although the evidence for their long-term clinical performance remains equivocal [1,2]. Zirconia as a restorative material has undergone significant development to optimise aesthetic results while being able to withstand masticatory forces [3]. Zirconia has also been suggested as a suitable material for endosseous implantation, as it has a superior soft-tissue response and lower affinity to bacterial biofilms [4] and may be an alternative option for patients who are allergic to titanium [5]. The use of zirconia may also circumvent the possibility that titanium wear particles exacerbate peri-implant mucosal inflammation, although conclusive evidence for this has yet to be demonstrated [6,7]. The higher risk of biomechanical failure due to fracture of zirconia implants should also be considered when substituting them for titanium (Ti) implants [8,9].

Until recently, literature involving zirconia implants focused on implants with a one-piece design [4] as it was considered difficult to incorporate the screw-joint mechanism with zirconia [10]. Single implant crowns on one-piece zirconia implants are usually cement-retained with an attendant increased risk of biological or biomechanical complications [4,11]. One-piece zirconia ceramic (ZC) implants with a sandblasted, large-grit, acid-etched surface and cemented crowns placed in single implant sites have shown a 97% success rate after 36 months [10]. Retrospective analysis of the failure rate of a two-piece ZC system was estimated at between 2% and 4% over a four-year period [12]. More recently, a prospective analysis of two-piece ZC implants supporting cement-retained single crowns demonstrated cumulative survival rates of 83% after six years, with biomechanical complications affecting 30.5% and biological complications affecting 8% of implants [13].

Although a body of literature is accumulating with regard to fixed prosthodontic restoration of both one-piece and two-piece ZC implants [14,15], more long-term studies are needed [3], and there is a paucity of clinical studies involving one-piece ZC implants supporting removable overdentures. A recent systematic review [16] that screened 2747 articles found only two articles that were suitable for inclusion and meta-analysis, both being published reports of the one-year outcomes for the patients included in the current 8-year follow-up [17,18].

The preclinical evidence supporting the use of ZC implants is also not extensive. A systematic review comparing ZC with Ti implants in various animal models over 30 years identified 29 published trials and concluded that the histological evidence for osseointegration of ZC implants was equivalent to Ti implants [19]. More recent studies comparing two-piece ZC and Ti systems in healed edentulous mandibular sites of dog or pig models, both unloaded [20,21] and loaded [22], also reported equivalency between ZC and Ti implants. A single report [23] has examined the response of one-piece ball-abutment ZC versus Ti implants in the femur and mandible of a preclinical sheep model; the current study extends this work and compares the results to implants with different configurations.

The aim of the current research was to (1) assess the clinical outcomes of ZC versus Ti one-piece implants supporting removable overdentures after eight years and (2) to assess the histological outcomes of the same ZC and Ti implants compared to Ti implants with different surface treatments, in an unloaded sheep animal model. The hypothesis was that ZC implants would show equivalent results to Ti implants, both clinically and histologically.

## 2. Materials and Methods

**Figure 1:**
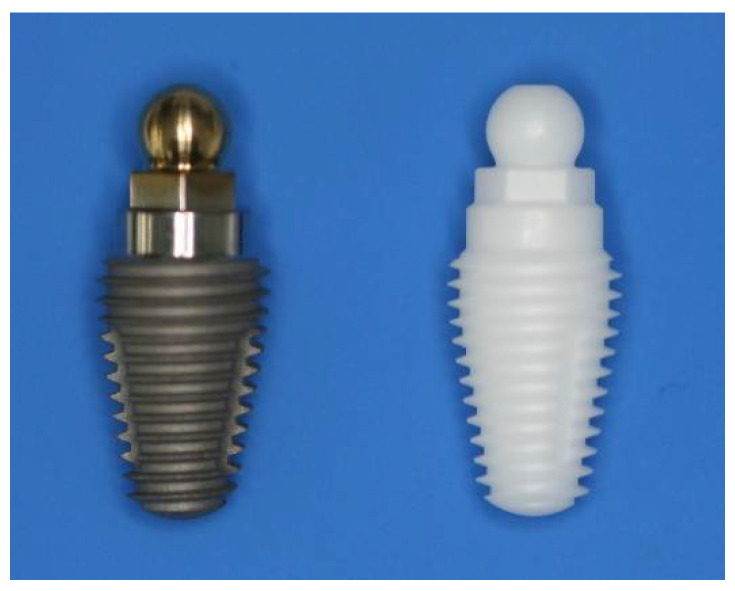
Images of the Ti (**left**) and ZC one-piece implants (**right**).

Implants:

The implants used in this study were identical one-piece ZC or Ti implants manufactured by Southern Implants (Irene, Centurion, South Africa) with a tapered threaded implant body, a transmucosal cylindrical collar, and a ball abutment (Figure 1). The ZC implants had an acid-treated surface with roughness Ra values of 0.5 to 0.8 μm; the Ti implants were acid-etched and sandblasted with surface Ra values of 1 to 2 μm. The diameters of the ball abutments were either 3.1 or 2.25 mm.

Clinical study:

The methodology and outcomes after one year have been reported previously [18,24] and were replicated at the 8-year recall. In brief, all investigations were carried out following the rules of the Declaration of Helsinki of 1975, and CONSORT guidelines for randomised trials were followed [25]. The study protocol was approved by the Regional Lower South Island Ethics Committee of New Zealand (LRS/09/06/023), and all participants provided signed informed consent. The trial was registered with the Australian New Zealand Clinical Trials Registry (ACTRN # 12610000037000).

At baseline [18], 24 participants were randomised into two groups of N = 12; two participants withdrew, and three died during the first year, leaving N = 8 (Ti group) and N = 11 (ZC group). Each participant received four implants in the maxilla and three implants in the mandible, with maxillary implants bilaterally in the premolar region and one off-centre in the incisal region and mandibular implants in the first molar regions and off-centre in the incisal region. An additional implant was placed in the mid-palate. Participants were provided with maxillary and mandibular implant overdentures using an unsplinted attachment system after 4 to 6 months of healing. Outcomes reported after 1 year included implant failure or survival (sub-categorised by implant material and by maxilla versus mandible), four-field table analysis according to Albrektsson and Zarb [26], and marginal bone level changes [24]; detailed analysis of surgical outcomes and peri-implant soft-tissue changes were reported separately [18].

Participants were re-examined at regular intervals for the following seven years. At eight years after baseline, all participants were recalled for an examination employing the original methodology. In brief, the presence or absence of implants was noted. For each of the six alveolar implants, soft-tissue parameters (probing pocket depth, recession, bleeding on probing) were recorded at four sites (mid-mesial, mid-distal, mid-buccal, and mid-lingual). Standardised digital intraoral radiographs and an ortho-pantomographic extra-oral radiograph were obtained. The distance from the top of the implant (head of patrix) to the first crestal bone contact at the mesial and distal aspect of the implant was measured as previously discussed.

Results were further categorised as follows:

1. Implant success was evaluated using four-field tables with the categories of success, survival, unaccounted for, and failure as per the criteria defined at the start of the trial, except that the palatal implants were defined as “surviving” since they could not have radiographic bone levels measured. Implants meeting the success criteria exhibited less than 1 mm bone loss during the first year and less than 2.4 mm over the subsequent seven years (<0.2 mm/year) and were free of mobility, pain, and neuropathy. Implants that were still functioning but did not meet the success criteria were considered as surviving. Implants in patients who dropped out of the study for any reason were considered unaccounted for. Deceased patients were included within the “unaccounted for” classification. An implant removed for any reason was considered a failure.
2. Peri-implant marginal bone levels were only recorded for the six alveolar implants; the palatal implants were excluded. Radiographs were evaluated using image analysis software (Image J 1.34S; National Institutes of Health, Bethesda, MD, USA). Measurements of mesial and distal surfaces for each group were averaged and compared statistically.
3. Peri-implant soft-tissue parameters, including probing depths, recession, and bleeding at four sites per implant, were only recorded for the six alveolar implants; the palatal implants were excluded. Pocket depths, recession, attachment loss, and percent bleeding scores were averaged for each group and compared statistically.

Biological complications for the alveolar implants (excluding palatal implants) were further classified as peri-implantitis based on the presence of bleeding and/or suppuration on probing, probing depths of ≥6 mm and/or bone levels ≥3 mm apical of the most coronal portion of the intra-osseous part of the implant [27].

Preclinical study:

The study was approved by the Animal Ethics Committee, University of Otago, Dunedin, New Zealand (Animal Ethics Committee number 30–11). Ten adult New Zealand Romney cross female sheep with an average weight of 65 kg were used in this trial.

**Figure 2:**
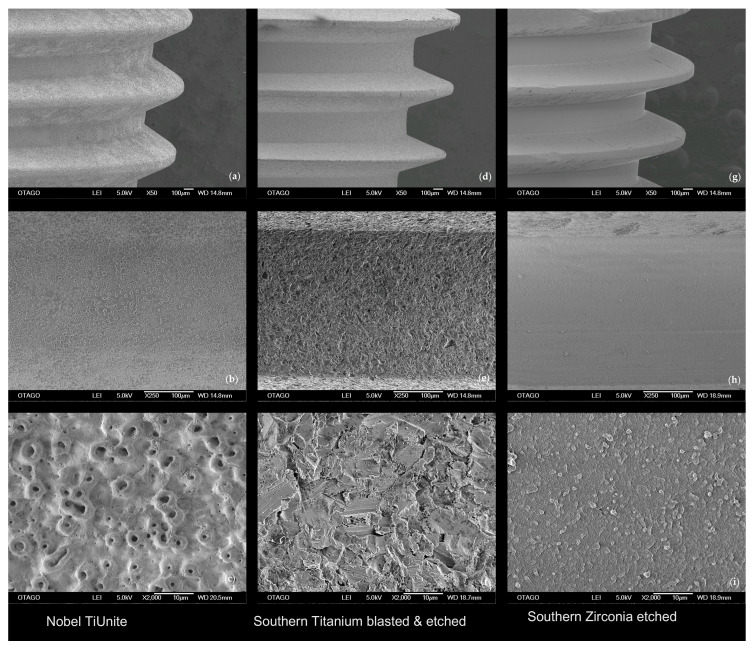
Scanning electron micrograph images of the three implant surfaces at, respectively, 50×, 250×, and 2000× magnification. (**a**–**c**) Nobel TiUnite™ anodised (**d**–**f**) Southern titanium blasted and etched, (**g**–**i**) Southern zirconia etched.

The methodology used for this study has been previously reported [18]. Briefly, one-piece ZC and Ti (including the ball abutment) implants as described above were installed into sheep femurs, along with two different types of Ti two-piece implants that were used as controls. Southern Implants ITC^®^ two-piece implants (Southern Implants, Irene, Centurion, South Africa) (ITC) were used. These have a thread and body configuration similar to the Straumann Standard Plus design, a turned collar of 1.8 mm height, an internal morse-taper and octagon connection, and a moderately rough (Sa = 1.43 µm) abraded surface of grade 4 Ti, obtained through sandblasting and chemical conditioning, which is identical to that of the test one-piece Ti ball-abutment implants. Additionally, two-piece implants with a different, anodised surface were also placed; these were Branemark System^®^ Mk III, 4.0 × 10 mm implants with TiUnite^™^ surfaces (NBC) (Nobel Biocare New Zealand Ltd., Auckland, New Zealand). Both of these two-piece systems were fitted with cover screws. Scanning electron micrograph images of the three different implant surfaces (Southern titanium blasted, Southern zirconia etched, Nobel TiUnite^™^ anodised) are shown in Figure 2.

## 3. Anaesthesia Technique for All Surgical Procedures

**Figure 3:**
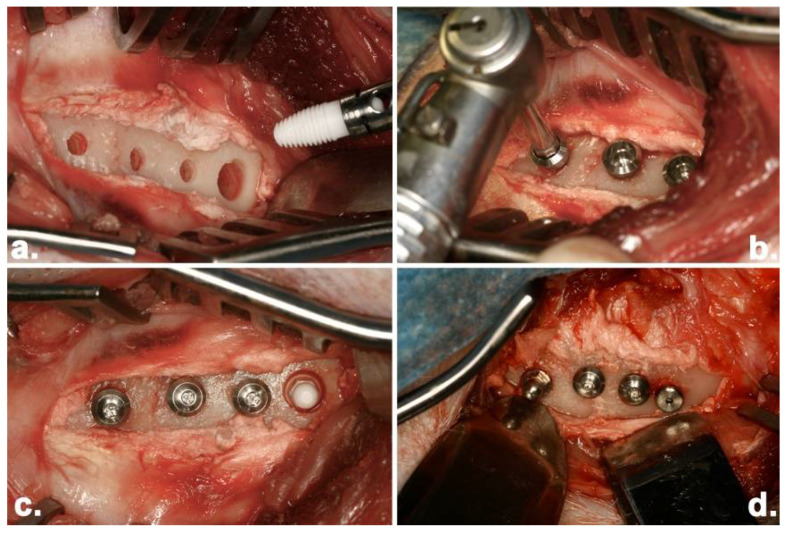
Surgical placement of dental implants into the left and right femurs of sheep. (**a**) placement of a Southern ZC one-piece implant; (**b**) placement of a Southern ITC two-piece implant; (**c**) after installation, from left to right, Southern ITC implants and Southern ZC one-piece implant (**d**) after installation, from left to right, Southern Ti one-piece implant, Southern ITC two-piece implants, NBC two-piece implant.

Animal surgery was carried out in the large animal operating theatre at the Hercus-Taieri Resource Unit (HTRU), University of Otago, New Zealand. The sheep were weighed and starved overnight prior to surgery and received preoperative antibiotics Trimethoprim (Amphoprim injection 1 mL/15 kg, Virbac New Zealand Ltd., East Tamaki, Auckland). General anaesthesia was induced using intravenous thiopentone 20 mg/kg (Bomac Laboratories Ltd., Manukau City, Auckland) and maintained by Halothane (1–2%) and nitrous oxide/oxygen (1:2). The surgical approach used the femoral dental implant model discussed by Chappard and colleagues (1999) [28] and further refined by Duncan et al. (2008) [29]. The hind legs were shaved, and the skin disinfected with iodine and alcohol. Each femur was exposed by a medial approach from the great trochanter to distal epiphysis via a skin incision of 6 to 8 cm in length. The periosteum was incised and raised, and osteotomies were prepared according to the manufacturer’s instructions. One of the one-piece implants (ZC or Ti) and one control implant (ITC or NBC) was placed alternatively into the left and right femurs. Additional modified ITC implants were also placed bilaterally and used for histological and reverse-torque experiments, which have been reported elsewhere. A total of 40 implants (N = 10 for each condition) were placed and reported in this study (Figure 3). The wounds were closed in layers with resorbable sutures.

### 3.1. Animal Euthanasia for Implants Retrieval

**Figure 4:**
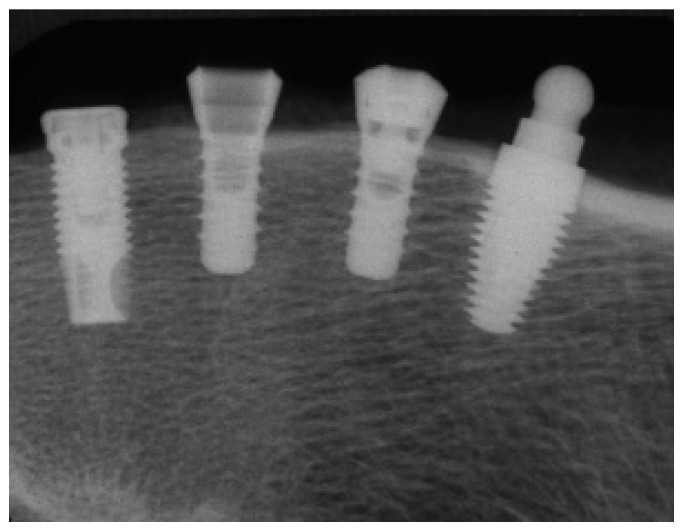
Radiograph of dissected sheep femur showing (left to right) Nobel implant, Southern ITC implants, Southern one-piece ZC implant.

After a healing period of 12 weeks, the animals were euthanised under general anaesthesia with an overdose of thiopentone, perfused via the carotid arteries with heparinised saline, followed by formalin fixative post fixed in formaldehyde for one week. They were radiographed (Figure 4), dissected out with fine handsaws using the radiograph as a guide, dehydrated in ascending grades of alcohol, cleared in xylol, and then infiltrated and embedded in methyl-methacrylate with di-butylphthalate as a plasticiser and benzoyl peroxide as an initiator (Sigma-Aldrich numbers M55909, 524980 and 517909, respectively, Sigma-Aldrich New Zealand Ltd., Auckland New Zealand) at approximately 10 °C using a standardised protocol [30,31] further modified in our lab [32]. Specimens were sectioned longitudinally using an R330 diamond wheel on a Struers Accutom-50® precision cut-off saw (Intellection Pty Ltd., Milton, Australia). Thick sections were glued to 3 mm-thick plastic slides using cyanoacrylate. Sections were then ground and polished to a final grit size of 4000 and final section thickness of 80–100 µm and stained with a solution containing one-part MacNeal’s tetrachrome (methylene blue, azur II, and methyl violet) and two parts toluidine blue. Sections were viewed using an Olympus Vanox-T microscope at 10× and 20× magnification (Olympus Australia Pty Ltd., Notting Hill, Australia), and digital images were captured using a Diagnostic Instruments SPOT RT Colour camera (SciTech Pty Ltd., Preston, Australia). The two most central sections from each implant were analysed. Bone-to-implant contact percentage (BIC%) was quantified histomorphometrically for the best three consecutive threads from each side of the implants using the NIH Image analysis software, ImageJ^®^ (ImageJ^®^-Research Services Branch, NIH, Bethesda, MD, USA—Rasband & ImageJ^®^, 1997–2012).

### 3.2. Statistical Analysis

Statistical software (version 25, SPSS, Inc. New York, NY, USA) was used in the analysis of the data. For the clinical study, histogram, stem-and-leaf plot, box plot, and measures of skewness and kurtosis, as well as Shapiro–Wilk and Kolmogorov–Smirnov tests, were used to assess the normality of quantitative data. Due to small sample sizes and non-normal distribution of the data, non-parametric statistics were used: radiographic data were compared using the Mann–Whitney U test, and peri-implant soft-tissue measures were compared using the Wilcoxon sign rank test, with the implant as the statistical unit. For the preclinical animal study, multiple pair-wise comparisons were made using Wilcoxon signed rank tests for BIC%. The levels of statistical significance were set at *p* < 0.05.

## 4. Results

**Clinical study**

**Figure 5:**
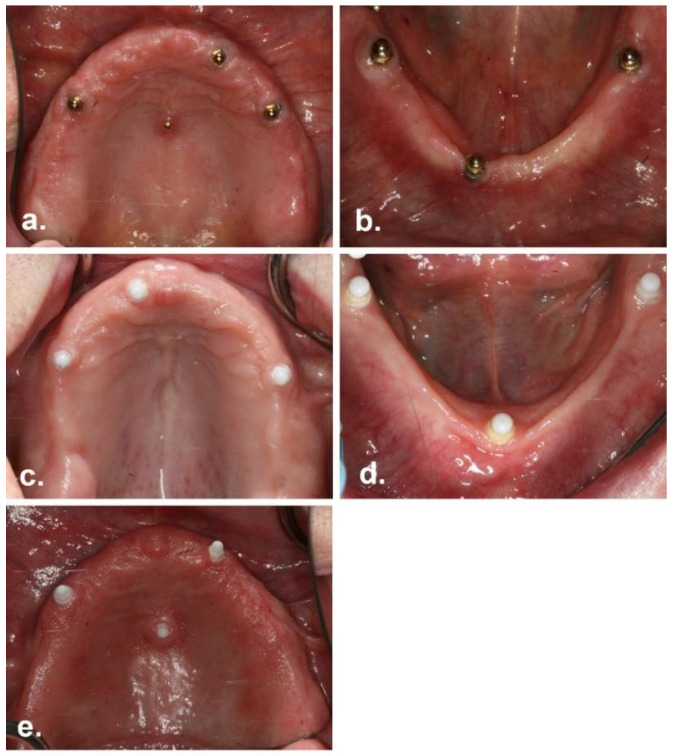
Representative images of the recalled participants. (**a**) Ti group, maxilla (**b**) Ti group, mandible (**c**) ZC group, maxilla; mid-palatal implant has failed (**d**) ZC group, mandible (**e**) ZC group, maxilla; the mid-palatal implant is present, but the posterior maxillary implant has failed.

Of the original 24 participants who commenced the trial, only eight could be recalled (33%). Of the remaining 16 participants, five are known to have died (21%), and 11 (46%) could not be contacted and were therefore classified as “unaccounted for”. For the Ti group, the distribution of “recalled”, “unaccounted for”, and “deceased” participants was equal (N = 4 for each); for the ZC group, N = 1 participant had died, N = 4 were recalled, and N = 7 were unaccounted for. The average age at the time of follow-up (excluding deceased participants) was 72 ± 11 years; the average age was 75 ± 8 years for those who attended recall and 70 ± 12 years for those who could not be contacted. All recalled ZC participants were male, and two Ti participants were male; overall, six male and two female participants were examined. Representative clinical images of the recalled participants are shown in Figure 5.

**Implant Success, Survival, and Failure compared with baseline**

After eight years, more than two-thirds of all the implants (67%) could not be unaccounted for (60% of Ti and 74% of ZC). Four-field table analysis of the remaining implants (Table 1) showed that the success rate for Ti implants (N = 16; 22.9%) was almost twice that of ZC implants (N = 10; 12.5%); conversely, the survival rate of Ti (10.0%) was half that of ZC (16.3%). Combining survival and success, the two groups were very similar after eight years (Ti 33%, ZC 29%).

## 5. Implant Outcomes in the Recalled Patients

The recalled patients had received a total of 56 implants (seven implants per participant), including one implant per person placed into an experimental site on the palate. Implant survival and failure after eight years was calculated for all implants in both groups and then considered just for the six implants per person placed into the maxillary (N = 3) and mandibular (N = 3) alveolar ridges, a total of N = 48 implants (Ti:N = 12 and ZC:N = 12).

Overall in the recalled patients, 11 implants had failed (19.6%). This consisted of five Ti and six ZC implants, of which three (37.5%) were palatal implants. The survival of implants is shown in Table 2. The overall survival rate for all implants in the recalled patients was 80.4%; excluding palatal implants, implant survival was 87.5% for Ti implants and 79% for ZC implants. All of the Ti alveolar implants failed in just one participant. Three of four participants with ZC implants lost one or more alveolar implants. All of the failed implants in both groups were maxillary implants. Two ZC implants (one palatal and one maxillary) had fractured off, leaving only the apex of the implant behind. No titanium implants fractured.

## 6. Peri-Implant Soft-Tissue Status

Recession for both Ti and ZC implants ranged from -3.0 mm to +3.0 mm, the latter representing mucosal overgrowth above the implant shoulder, sometimes covering the ball abutment. Mean recession for Ti implants (0.3 ± 0.9 mm) was not significantly different from ZC implants (0.4 ± 1.0 mm, *p* = 0.6). Pocket depths ranged from 1.0 to 4.0 mm (Ti) and 0.0 to 6.0 mm (ZC). Mean pocket depth for Ti implants (2.2 ± 0.6 mm) were significantly deeper than for ZC (1.9 ± 0.7 mm, *p* = 0.04). Mean attachment loss of 1.9 ± 0.6 mm for Ti implants was significantly greater than 1.4 ± 0.7 mm for ZC implants (*p* = 0.02).

There was a little difference in the average number of sites that bled on probing (18% for Ti, 22% for ZC) but there were marked variations between individuals ranging from 0% to 83% for the Ti group and 5% to 40% for the ZC group. The participant who lost all three maxillary implants had bleeding at 10 of 12 sites (83%) around the three remaining mandibular implants, and the participant who lost two maxillary ZC implants had a bleeding score of 40% around the remaining implants. Peri-implant soft-tissue measurements are shown in Table 3.

## 7. Radiographic Bone Level

**Figure 6:**
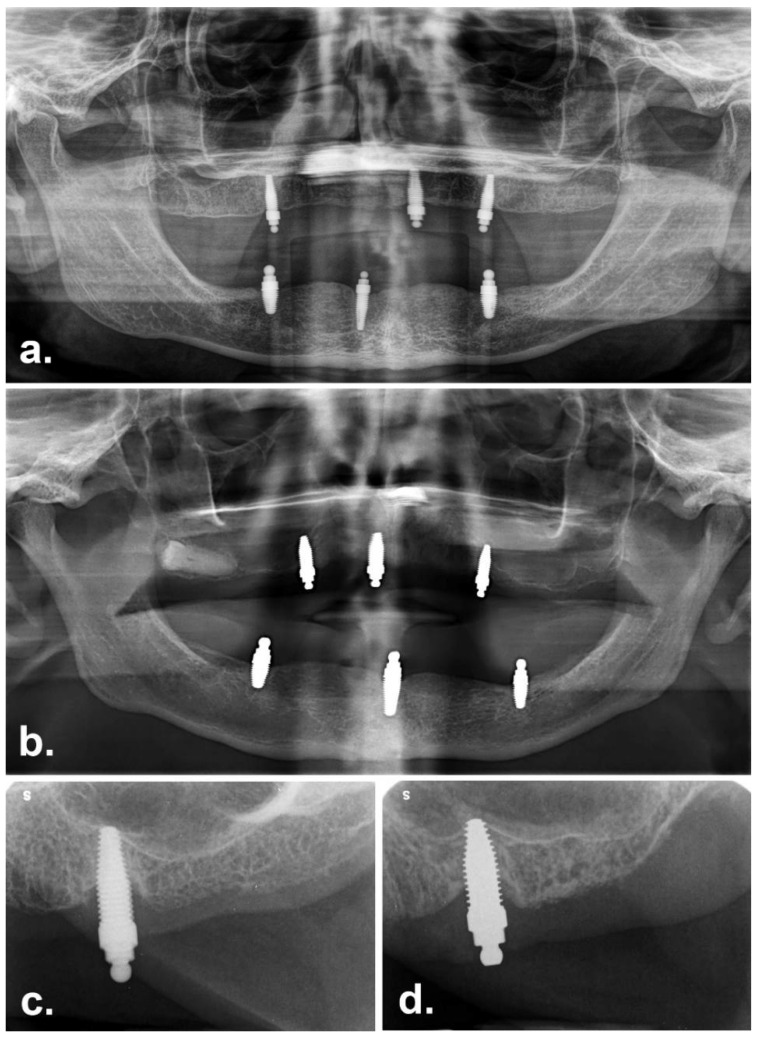
Representative radiographs from the recalled participants: (**a**) Orthopantomogram of participant with Ti implants (clinical images in Figure 4) (**b**) Orthopantomogram of participant with ZC implants (clinical images in Figure 4) (**c**) intraoral peri-apical radiograph showing peri-implant bone loss around a Ti implant in the maxilla (**d**) intraoral peri-apical radiograph showing peri-implant bone loss around a ZC implant in the maxilla.

After eight years, the Ti group exhibited a mean radiographic bone loss of 1.67 ± 1.58 mm (recorded for 4 patients with 22 implants). For the ZC group, one participant was not radiographed due to a medical event that occurred at the review appt; the mean radiographic bone loss for this group was 1.60 ± 1.35 mm (3 patients with 14 implants). Representative radiographs from the recalled participants are shown in Figure 6.

Overall, only one participant in each group showed no evidence of peri-implantitis and had not lost any alveolar implants. For the surviving implants, 57% of Ti and 52% of ZC implants showed evidence of peri-implantitis. When the number of implants showing peri-implantitis and the number of implants that had failed were combined into a single figure for ZC versus Ti, the outcomes were the same for both groups; only 37.5% of alveolar implants had neither failed nor showed evidence of peri-implantitis.

For the palatal implants, two of four Ti implants had been lost, and one of three ZC implants had failed. However, all of the remaining implants showed evidence of peri-implantitis.

## 8. Preclinical Study

All animals survived surgery and were available for evaluation. No local infection or pathology was noted around any implants. The overall implant survival rate was 100%.

## 9. Histomorphometry

**Figure 7:**
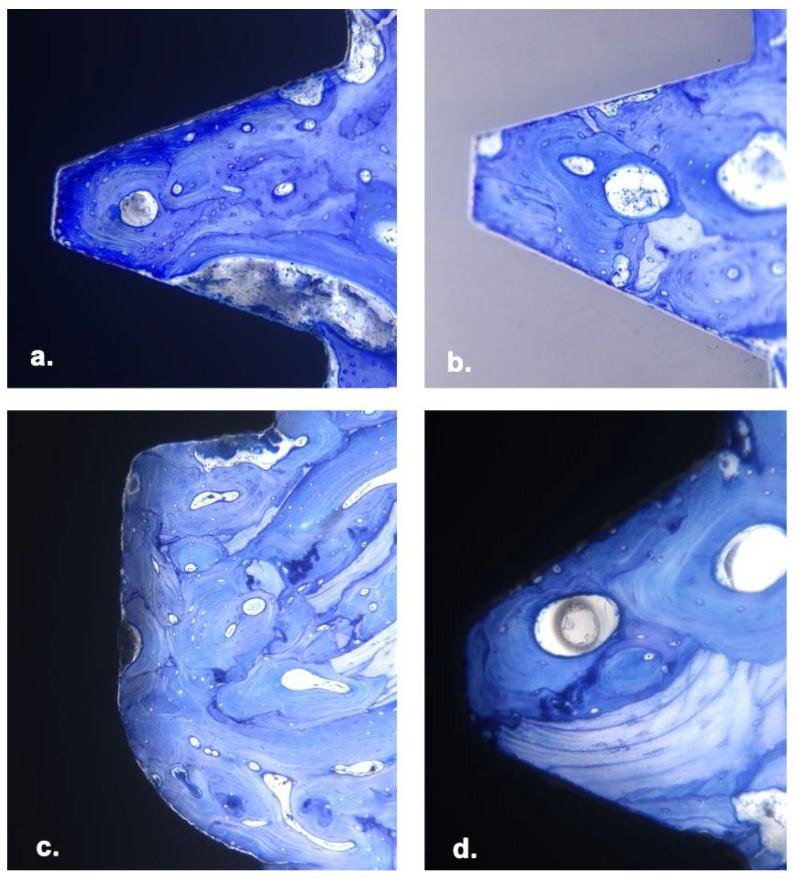
Histological images of implants in sheep femur 20× magnification, stained with MacNeil’s Tetrachrome and Toluidine blue. (**a**) Southern Titanium one-piece (**b**) Southern Zirconia one-piece (**c**) Southern ITC two-piece (**d**) Nobel two-piece implant.

Mean BIC% is shown in Table 4, and histological images are shown in Figure 7. The BIC% was slightly higher for ZC compared with Ti one-piece implants, but this difference was not statistically significant (80.0% vs. 75.7%, *p* = 0.3). The BIC% for the ITC implants, a two-piece system with the same blasted and etched surface as the Ti one-piece implants but with a different thread configuration, did not differ significantly from either the ZC or Ti implants (75.1%, *p* = 0.2 and 0.7, respectively). The NBC two-piece implants, which have a similar thread configuration to the two Southern one-piece implants (ZC and TI) but a different, anodised (TiUnite^™^) surface, had the highest BIC%, although this differed significantly from the ITC implants only (84.2%, *p* = 0.4).

## 10. Discussion

This study reports the outcomes after eight years for a randomised controlled trial in completely edentulous participants that compared a novel design of ZC one-piece ball-abutment implants with an etched surface against Ti one-piece ball-abutment implants with a blasted and etched surface; all participants received removable implant overdentures in the maxilla and mandible. In addition, this paper reports the short-term histological outcomes of the same one-piece ZC and Ti implants, installed for 12 weeks and never loaded, in a sheep femur model. Outcomes for bone-to-implant contact are contrasted against titanium implants with either a similar surface but different thread macro-design or with a similar thread macro-design but markedly different (anodised) surface.

One-year results for the original clinical trial [25] were reported for 19 of the original 24 participants. For the three implants placed into conventional positions on the maxillary alveolar ridge, nine Ti (28.1%) and 18 ZC implants (45%) failed after 1 year. Of the three implants placed into the mandible of the same participants, one (1.8%) Ti and three (5.3%) ZC implants failed. In the current study, eight of the original participants were re-examined after eight years; the results were broadly in line with the original one-year findings. Although there had been considerable drop-out from the trial and thus a high percentage of implants that could not be accounted for, of the three implants placed into the maxillary alveolus in eight patients, three (12.5%) Ti and five (20.8%) ZC had failed after eight years. For the mandible, after eight years, no (0%) Ti or ZC implants failed.

In the initial report, after one year [18], 66.7% of Ti implants placed into alveolar ridges fulfilled the “success” criteria versus 67.6% of ZC implants. After eight years, we found that 57.1% of Ti versus 41.7% of ZC implants fulfilled the cumulative “success” criteria, but more than two-thirds of all the implants (67%) could not be unaccounted for (60% of Ti and 74% of ZC). Four-field table analysis of the remaining implants (Table 1) showed that the success rate for Ti (N = 16; 22.9%) was almost twice that of ZC (N = 10; 12.5%); conversely, the survival rate of Ti (10.0%) was half that of ZC (16.3%). Combining survival and success, the two groups were very similar after eight years (Ti: 33%, ZC: 29%). However, when the data are considered for the eight recalled patients in isolation, twice as many Ti implants were successful as surviving (57.1% versus 25%), whereas fewer ZC implants were successful than surviving (35.7% versus 46.4%). The failure rate was equivalent for both groups. In the initial one-year reports [24,25], most implant failures occurred in the maxilla; similarly, after eight years, we found that all the failures had occurred in the maxilla.

After one year, mean probing depths around Ti and ZC implants were both 2.2 mm; after eight years, mean pockets depths around Ti implants were still 2.2 mm, while around ZC implants, these were significantly reduced (1.9 mm, *p* = 0.04). Mean recession around Ti and ZC implants (0.9 and 0.7 mm, respectively) had reduced by eight years to 0.3 mm and 0.4 mm, respectively. There was no difference in bleeding scores between the two groups after eight years. Mean bone levels showed a marked decline, from a mean of 0.18 mm for Ti and 0.42 mm in ZC implants after one year to 1.7 mm and 1.6 mm in Ti and ZC implants, respectively, after eight years. However, the difference between the two implant materials was no longer statistically significant. A grouping phenomenon could be observed, suggesting that in some participants, peri-implant health and crestal bone loss played a role in implant failure, whereas in others, this was not the case; these phenomena seemed to be independent of the implant material. Conversely, the grouping of implant failures in the maxilla rather than mandible for both implant materials suggests that implant failures were more related to an interaction between biomechanical factors and bone quality.

Previous reports for one-piece ZC implants with cement-retained single crowns gave a 97% success rate [10]). In this study, the average age of participants was 48 years old (range 18–78 years old), and 28% of the study group was excluded from follow-up after just 3 years. Participants were dentate with a single tooth edentulous space bounded by extant teeth, which implies significantly different biomechanical forces compared to the completely edentulous participants in the current study. Patients with periodontitis were excluded, which was not possible with the edentulous participants in the current trial; however, it is known that older edentulous patients continue to harbour periodontal pathogens in mucosal sites after full dental extractions [33,34] and that an increase in pathogens is associated with denture wearing [35]. Others have reported that ZC implants in patients previously treated for periodontitis showed equivalent outcomes to periodontally healthy patients (success rate 94% versus 95%) after one year in function [36].

The experimental palatal implant site performed poorly. Of the eight implants, three (37%) failed, and all the remaining implants showed inflammation and peri-implant pockets. While this biological complication could not be diagnosed as “peri-implantitis” as it was not possible to visualise bone loss using intraoral radiographs, this palatal site could not be recommended for supporting removable maxillary implant dentures.

The short-term histomorphometric outcomes in a preclinical animal model for these implants suggested a different story from the long-term clinical outcomes. ZC implants had equivalent bone-to-implant contact to Ti implants of the same design when allowed to heal unloaded in trabecular bone, despite the zirconia implants having a less developed and roughened surface. Changing the implant morphology (different thread design, two-piece rather than one-piece) did not enhance the initial bone-implant contact. The oxidised TiUnite^™^ surface on Ti implants is an additive rather than subtractive process (such as is seen with sandblasting and etching). It has previously been shown that this anodisation process enhances osseointegration of Ti alloy implants in sheep femurs [29] and sheep mandible [37] but not sheep maxillary sinuses [38], and is also effective when applied to titanium-zirconium alloy tested in sheep femurs [39]. An equivalent additive and porous surface to TiUnite^™^ has been produced on ZC implants and called ZiUnite^™^ [40]; histomorphometric analysis comparing two-piece Ti implants with TiUnite^™^ against ZC with ZiUnite^™^ in rabbit femoral condyles after three and six weeks found equivalent results (%BIC, ZC vs. Ti; 3 weeks: 70.5% vs. 77.6%, 6 weeks: 69.7% vs. 67.1%). ZC implants without surface modifications were not included in this study. Furthermore, a clinical study where the ZiUnite^™^ surface was incorporated onto one-piece implants found increased crestal bone loss ≥2 mm after one and three years, and these implants could not be recommended for clinical use [41,42].

The sheep femoral site consists of relatively soft trabecular bone and might be considered a suitable model for the bone found in edentulous maxillary alveolar sites in human patients. However, our findings of high BIC% in the sheep epicondyle site contrast with the current clinical results for ZC implants, especially in the maxilla under loaded conditions over time. The most likely explanations for this seem to be the unavoidable immediate loading experienced during the healing phase, as the one-piece design does not allow for two-stage implant surgery.

A systematic review and meta-analysis of ZC versus Ti implants in animal models found no significant difference in BIC% values when comparing machined-surface Ti and ZC or comparing sandblasted Ti and ZC implants, although some reports suggested that unmodified ZC implants showed significantly better BIC% than sandblasted ZC implants [43]. A comprehensive review of ZC implants in animals reported 30 papers covering 29 trials [19]; just over half comprised large animal models (three in dogs, four in sheep, eight in pigs, and one in primates), and two-thirds were non-oral sites. After 12 weeks of unloaded healing in the sheep femur, the acid-etched one-piece ZC versus sandblasted, acid-etched one-piece Ti implants showed a mean BIC% of 80% for ZC versus 76% for Ti implants in the current study. Results in sheep models comparable to the findings in the current study include our previous work on femur and mandible after 12 weeks (ZC vs. Ti BIC%: femur: 85.5% vs. 79%; mandible: 72% vs. 60%) [23] and sandblasted Ti versus ZC implants in tibia after 12 weeks (ZC vs. TI: 75% vs. 82%) [44]. Others reported results for sandblasted and etched Ti versus ZC implants in sheep iliac bone after 6 weeks (ZC vs. Ti: 75% vs. 80%) [45].

BIC% in other large animal models with comparable healing periods varied from 59% for ZC to 41% for Ti implants in dogs [46,47] and 45% to 71% for ZC versus 35% to 83% for Ti implants in pigs [48,49,50]. An older study in primates with sandblasted custom-made one-piece implants, integrated for three months and then loaded with cemented crowns for five months, found a mean BIC% of 67% for ZC versus 73% for Ti implants [51]. It is notable, however, that a recent review did not find that the surface structure of ZC implants influenced BIC% but did find that the chosen animal model had a significant influence on outcomes [52].

More recently, two-piece tissue-level ZC implants with a sandblasted and etched surface referred to as ZLA (equivalent to the Straumann SLA surface) were compared to matching Ti implants in healed edentulous posterior sites in dog mandibles [22]. After 6 weeks of healing, implants were loaded for 4 or 16 weeks. For those integrated for a total of 22 weeks, the mean BIC% was 71% for ZC versus 70% for Ti implants. Subsequently, Thomé et al. (2021) compared unloaded two-piece sandblasted and acid-etched ZC versus Ti implants in the healed posterior edentulous mandible of minipigs [20]. Mean BIC% after 8 weeks was 78% for ZC vs. 81% for Ti implants. Chacun et al. (2021) compared unloaded two-piece sandblasted and acid-etched ZC versus Ti implants in the healed edentulous mandible of dogs for 4 or 13 weeks. Mean %BIC after 13 weeks was reported as 86% for ZC vs. 92% for Ti implants [21].

Our study suffered from several limitations. The clinical arm was based on older-aged, completely edentulous participants, and it proved very difficult to recall them after eight years. The limitations of single-piece implants with patrices are well-known, and the mechanical abrasion of zirconia against matrices resulting in reduced denture retention may have resulted in adverse forces that manifested as fractured zirconia implants and adverse biological outcomes; this is recognised as a significant issue for older denture-wearing patients [53]. Furthermore, results in edentulous older-aged participants loaded with unsplinted implant overdentures are not generalisable to (for example) middle-aged dentate participants restored with single-tooth implants or short-span fixed bridges. However, within these limitations and in this specific population, the ZC implants showed equivalent outcomes to the Ti implants. The preclinical study provided additional information regarding the early osseointegration events after implant placement, but as always, results derived from short-term, unloaded, non-oral sites in an animal species should be interpreted with caution with respect to potential outcomes in human patients. When relating histological evidence for osseous healing around dental implants in preclinical animal models to the possible outcome of equivalent implants in human patients, we must remain conscious of inter-species differences with respect to the development of bone at different anatomical sites (particularly long bone versus mandibular sites); bone size and macrostructure; histological microstructure; elemental composition; remodelling rates and animal lifespan [54]. While no one species or animal model completely recapitulates implant healing in human bone, provided the researcher acknowledges the effect of differences in bone architecture and remodelling, various non-human species can provide useful information during the testing and development of bone-implant materials [54,55]. However, as demonstrated in our study, understanding the long-term clinical success of new materials intended for dental implants rests upon the results of long-term randomised clinical trials in human participants.

## 11. Conclusions

The short-term outcomes in an animal model were encouraging, with both ZC and Ti one-piece implants showing excellent bone-implant contact in unloaded femoral sites. The long-term clinical results presented a somewhat different picture. Outcomes for implants placed into conventional alveolar ridge sites were equivalent for both groups, but more than two-thirds of the implants had either failed or showed evidence of peri-implantitis. Implants placed into mid-palatal sites demonstrated poorer outcomes, a high failure rate, and significant inflammation and peri-implant pockets around remaining implants. The palatal site cannot be recommended for supporting maxillary removable implant overdentures. While ZC and Ti implants showed equivalent clinical performance in this study, the risk of peri-implantitis and implant loss in older, completely edentulous patients remain a significant consideration.

## Figures and Tables

**Table 1:** Implant outcome: four-field table analysis (Albrektsson and Zarb, 1993).

Treatment Group	Success No. (%) ± *	Survival No. (%)	Unaccounted for No. (%)	Failure No. (%)
Ti group (control)	16 (22.9)	9 (10)	42 (60)	5 (7.1)
ZC group (test)	10 (12.5)	13 (16.3)	59 (73.8)	5 (6.3)

* Calculation of percentages based on original group sizes in Table 14 from Osman et al. 2014.

**Table 2:** Distribution of failed implants in recalled patients.

	Maxillary	Mandibular	Combined
Titanium			
Alveolar	3	-	3
Palatal	2	-	2
*Total*			5
Zirconia			
Alveolar	5	-	5
Palatal	1	-	1
*Total*			6
Combined			
	8	-	8
	3	-	3
			11

**Table 3:** Peri-implant soft-tissue measurements.

	Ti	ZC	*p*
Mean pocket depths (mm) [SD]	2.2 [0.6]	1.9 [0.7]	0.04
Mean recession (mm) [SD]	0.3 [0.9]	0.4 [1.0]	0.6
Mean attachment loss (mm) [SD]	1.9 [0.6]	1.4 [0.7]	0.02
Mean bleeding score (%) [range]	18 [0–83]	22 [5–40]	NS

**Table 4:** Mean BIC% for implants.

Implant Type	N	BIC% [SD]
Southern Zirconia one-piece (ZC)	10	80.0 [17.4]
Southern Titanium one-piece (TI)	10	75.7 [20.6]
Southern Titanium two-piece (ITC)	10	75.1 [16.1]
Nobel Titanium two-piece (NBC)	10	84.2 [16.7]

## Data Availability

Data are available on request from the authors.
